# A Determination of p97/VCP (Valosin Containing Protein) and SVIP (Small VCP Interacting Protein) Expression Patterns in Human Testis

**DOI:** 10.3390/medicina59061079

**Published:** 2023-06-03

**Authors:** Akgül Arıcı, Fikret Erdemir

**Affiliations:** 1 Department of Medical Pathology, Tokat Gaziosmanpasa University, 60100 Tokat, Turkey; 2 Department of Urology, Tokat Gaziosmanpasa University, 60100 Tokat, Turkey

**Keywords:** p97/VCP, SVIP, UPS, human testis

## Abstract

*Background and Objectives*: The ubiquitin proteosome system (UPS) is a non-lysosomal pathway that functions in all eukaryotes. The transport of polyubiquitinated proteins to proteosomes takes place via the p97/Valosin-containing protein (VCP) chaperone protein. The p97/VCP binds to polyubiquitinated proteins, allowing these proteins to reach the proteasome and, thus, their destruction. In the case of p97/VCP deficiency, ubiquitinated proteins accumulate in the cell cytoplasm, and their subsequent failure to break down produces various pathological conditions. Small VCP interacting protein (SVIP) and p97/VCP proteins have not been studied in human testicular tissues from different postnatal periods. Therefore, in our study, we aimed to examine the expression of SVIP and p97/VCP in postnatal human testicular tissues. Our study aimed to contribute to further studies on the use of these proteins as testicular cell biomarkers in cases of unexplained male infertility. *Materials and Methods*: Immunohistochemical studies with the aim of determining the expression of p97/VCP and SVIP proteins in neonatal, prepubertal, pubertal, adult, and geriatric human testis tissues were performed. *Results*: In testicular sections obtained from a neonatal group, p97/VCP and SVIP were localized in different testicular and interstitial cells, and the lowest expression was observed in this group. While the expressions of these proteins were low in the neonatal period, they increased gradually in the prepubertal, pubertal and adult periods. The expression of p97/VCP and SVIP, which peaked in adulthood, showed a significant decrease in the geriatric period. *Conclusions*: As a result, the expression of p97/VCP and SVIP correlated with the increase in age, but it decreased significantly in older groups.

## 1. Introduction

There are two major pathways for the degradation of proteins in eukaryotic cells. One of these is lysosomal proteolysis in the acidic environment of the lysosome, and the other is the ubiquitin–proteasome degradation pathway. Ubiquitin is a polypeptide consisting of 76 amino acids. Ubiquitin binds to the amino group of the lysine side chains of a protein, marking that protein for degradation. At this point, new ubiquitins are added, forming a multiubiquitin chain. The protein labeled with multiple ubiquitins is degraded by the proteasome, a protease complex composed of multiple subunits. Ubiquitins are released for reuse. ATP energy is used for ubiquitin binding and the degradation of the labeled protein. The binding of ubiquitins to the protein to be degraded (ubiquitination) occurs with the help of the ubiquitin-activating enzyme (E1), ubiquitin-binding enzyme (E2) and recognition protein (E3) [1,2,3].

The transport of polyubiquitinated proteins to proteosomes takes place via the p97/Valosin-containing protein (VCP) chaperone protein. It has been shown that p97/VCP physically binds directly to both the proteasome and polyubiquitin substrates and the polyubiquitinated chains [4]. p97/VCP is a protein in the ATPase family and is one of the most highly expressed proteins in humans. p97/VCP is mainly localized in the endoplasmic reticulum (ER) [5]. p97/VCP is important in protein metabolism and intracellular hemostasis. It supports the degradation, refolding, recycling, and displacement of target proteins [6,7]. p97/VCP plays a critical role in a wide variety of cellular processes through the binding of different cofactors and adapter proteins. These processes include protein quality control (ER-associated degradation, mitochondria-associated degradation, ribosome-associated degradation, and proteasomal degradation), cell cycle, cell death (apoptosis and autophagy), chromatin-associated degradation, Golgi assembly, endosomal trafficking, and lipid droplet biogenesis. In the case of p97/VCP deficiency, ubiquitinated proteins accumulate in the cell cytoplasm, and their subsequent failure to break down produces various pathological conditions [8,9,10].

ER is an organelle in which proteins and lipids are synthesized and undergo changes after synthesis. The chaperones in the ER lumen ensure that these newly synthesized proteins are properly folded. They also prevent the accumulation of misfolded proteins by the ER-associated degradation pathway (ERAD) [11]. ERAD enables the proteasomal degradation of misfolded proteins. This degradation consists of several important steps such as recognition, ubiquitination, retrotranslocalization, deglycosylation, and the transport of the substrate to be cleaved to the proteasome [12]. p97/VCP is a protein that removes misfolded proteins from the ER and enables proteasomal degradation. p97/VCP interacts with many adapter proteins and cofactors and displays different functions in the cell. These interactions are due to specific protein domains present in both VCP and cofactors. The VCP-interacting motif (VIM-VCP interacting motif), which is one of these domains, is found in SVIP and gp78 (ubiquitin ligase) proteins [13,14].

The small VCP interacting protein (SVIP) is an adapter ER protein that can bind directly to p97/VCP. It is localized on the cytosolic surface of the ER membrane. SVIP inhibits ERAD by interacting with p97/VCP. It achieves this inhibition by preventing the formation of the gp78-p97/VCP-Derlin1 complex [12]. Therefore, the use of SVIP as an inhibitor in cellular conditions or pathologies in which ERAD is over-activated may lead to the normal course of many signaling pathways and may protect the stressed cell (in the case of fragmented ER, ER detached from ribosomes, the formation of lipid droplets, etc.) [11,12].

Studies have shown that SVIP is localized in the central and peripheral nervous system [15]. Moreover, SVIP and VCP have been found to exhibit colocalization in the neuronal cell body. Studies on the expression of SVIP in other cells and tissues are limited. However, the expression of p97/VCP and its interacting proteins in developing rat testes has been demonstrated in immunohistochemical and Western blot studies. In experimental studies, it has been reported that these proteins have roles in spermatogenesis, ischemia–reperfusion, Sertoli cell function or development of testis and epididymis [16,17].

Studies on the expression and functions of SVIP and p97/VCP in human testicular tissue are few. As far as we know, we have not encountered studies in the literature in which the expressions of p97/VCP and SVIP proteins were described by an immunohistochemical method in normal human testicular tissues of different postnatal age periods.

Therefore, our study aims to examine SVIP and p97/VCP protein expressions using an immunohistochemical staining method in human testicular tissues at different periods of postnatal life. In addition, cellular (Sertoli, Leydig, germ cells, testicular macrophages) expression differences in testicular tissue were evaluated.

## 2. Materials and Methods

The study was carried out with the approval of Tokat Gaziosmanpaşa University Faculty of Medicine Clinical Research Ethics Committee and the rules of the Helsinki Declaration (No:15-KAEK-159). Cases where patients underwent orchiectomy due to diagnoses such as testicular tumor, testicular torsion and atrophic testis between January 2006 and December 2015 were retrospectively scanned and analyzed by taking paraffin blocks from the Gaziosmanpasa University Hospital Pathology Department archive. The cases were divided into five groups according to their ages: neonatal (0–6 months), prepubertal (1–11 years), pubertal (12–18 years), adult (19–64 years) and geriatric (65+ years). Six cases for each group were included in the study. SVIP and p97/VCP expressions were determined with immunohistochemical studies on five micrometer-thick sections prepared from paraffin blocks containing normal testicular tissue from all these cases. Protein expressions in testis tissues of different ages were evaluated with H-SCORE analysis for each protein and analyzed with appropriate statistical methods.

### 2.1. Immunohistochemistry

Five-micron sections taken with a microtome from testicular tissues embedded in paraffin were passed through xylol and graded alcohol series, and then boiled in the microwave 2 times for 5 min in 0.1 mM citric acid solution (pH: 6). After washing with phosphate-buffered saline (PBS) 3 times for 5 min, endogenous peroxidase activity was suppressed with 3% hydrogen peroxidase. After washing again with PBS 3 times for 5 min, the sections were blocked with blocking serum (ScyTek Laboratories, Logan, UT, USA) for 10 min at room temperature to prevent non-specific reaction. After 10 min of incubation, the block solution was removed from the wash and primary antibodies were applied: mouse monoclonal p97/VCP (ab11433, 1:500, Abcam, Cambridge, UK) and rabbit monoclonal SVIP (HPA039807, 1:50, Sigma, Darmstadt, Germany). Primary antibodies were incubated with tissues overnight at 4 °C. Sections treated with primary antibodies were washed with PBS at room temperature and then incubated with biotinylated secondary antibodies (ScyTek Laboratories, Logan, UT, USA) and peroxidase-labeled streptavidin (ScyTek Laboratories, Logan, UT, USA) for a total of 50 min, respectively. Peroxidase activity was made visible after incubation with 3-amino-9-ethylcarbazole (AEC) (ScyTek Laboratories, Logan, UT, USA) chromogen and counterstained with Mayer’s hematoxylin (ScyTek Laboratories, Logan, UT, USA). The slides were then sealed with water-based sealing solution (Fisher Chemicals, Springfield, NJ, USA). For controls, sections were incubated with a similar concentration of normal mouse or rabbit serum as the primary antibody. Stained sections were photographed under a Leica microscope (Leica DM2500, Nussloch, Germany).

### 2.2. H-SCORE and Statistical Analysis

The evaluation of the immunohistochemical labeling was performed using H-SCORE analyses as described previously [16]. The intensities of p97/VCP and SVIP immunoreactivities were semiquantitatively evaluated using the following intensity categories: 0 (no staining), +1 (weak but detectable staining), +2 (moderate or distinct staining), and +3 (strong staining). For each tissue, an H-SCORE value was derived by calculating the sum of the percentages of cells that were stained at each intensity category and multiplying that value by the weighted intensity of the staining using the formula H-SCORE: P Pi (i þ l), where ‘‘i’’ represents the intensity scores and ‘‘Pi’’ is the corresponding percentage of the cells. In each slide, 5 randomly selected areas were evaluated under a light microscope (×40 objective), and the percentage of cells for each intensity within these areas was determined at different times by 2 investigators who were not informed about the type and source of the tissues. The average score of both observers was used. The H-SCORE values were shown with a graph.

Statistical analyses were carried out using Sigma Stat version 3.5 (Jandel Scientific Corp., San Rafael, CA, USA) and significance was evaluated as *p* < 0.05. Possible differences in tissues obtained at different postnatal periods were evaluated with comparative tests (ANOVA, *t*-test) and correlation regression analyses (Pearson, Spearman).

## 3. Results

The mean age of the cases was 23.86 ± 26.49 years (neonatal 0.31 ± 0.17, prepubertal 4.33 ± 2.58, pubertal 14.17 ± 1.47, adult 29.33 ± 6.77 and geriatric 71.17 ± 7.6).

The evaluation of the cellular expression of p97/VCP and SVIP in neonatal, prepubertal, pubertal, adult and geriatric human testicular tissues was carried out using immunohistochemistry.

**Figure 1:**
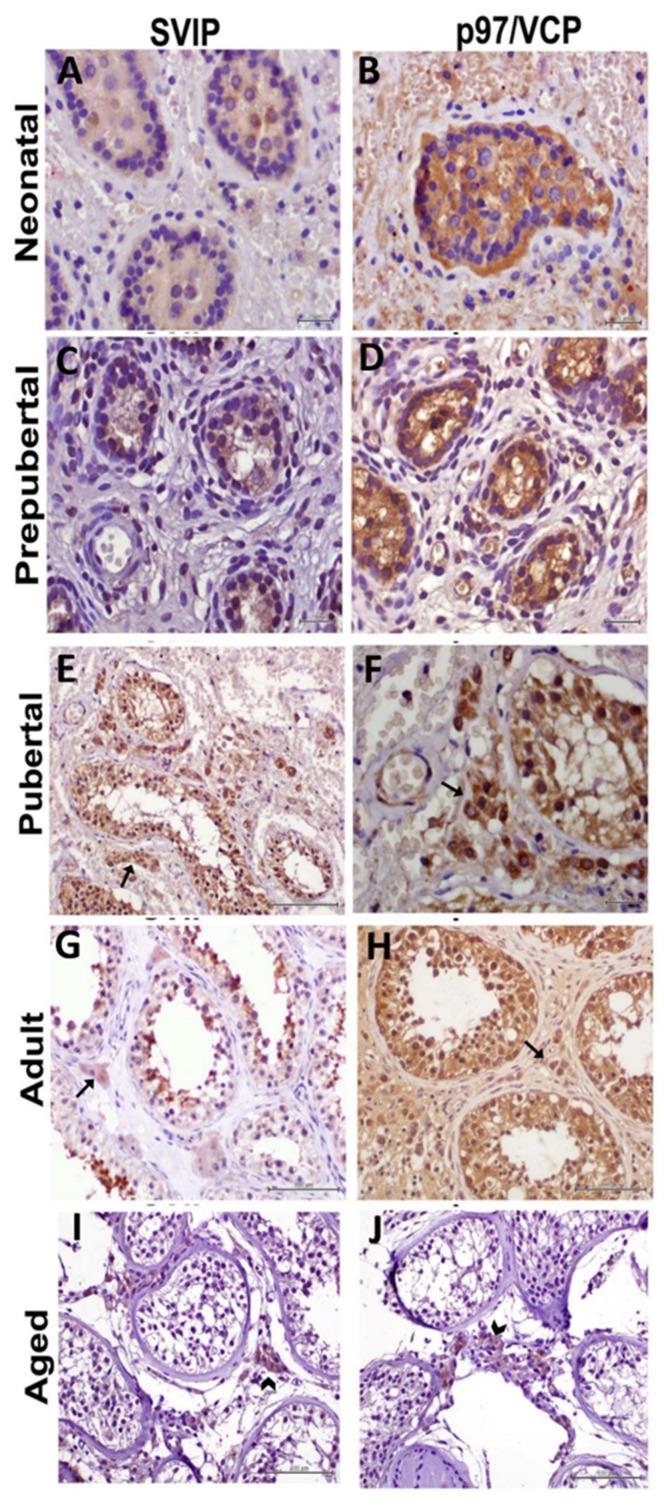
Immunohistochemical detections of SVIP in the testes in neonatal, prepubertal, pubertal, adult and aged groups (**A**,**C**,**E**,**G**,**I**). Immunohistochemical detections of p97/VCP in the testes at neonatal, prepubertal, pubertal, adult and aged groups (**B**,**D**,**F**,**H**,**J**). Leydig cells were notably present in (**E**,**F**,**G**,**H)** (arrow). Connective tissue and Leydig cell aggregations were notably present in (**I**,**J**) (arrowhead).

The localization of p97/VCP and SVIP in different testicular and interstitial cells in testicular sections obtained from patients belonging to the neonatal group was demonstrated. In this group, where the tubule wall was quite thin, a thin row of germ cells was observed just below the wall. In the SVIP protein sections, weak staining was observed in the peritubular and tubular areas. With SVIP, much less staining was seen in the peritubular area than in the tubular area. Weak to moderate p97/VCP immunostaining was observed in tubule wall, germ cells, and peritubular area. With p97/VCP, less staining was seen in the peritubular area than in the tubular area (Figure 1A,B).

It was observed that the number of cells in the peritubular and tubular areas increased in the prepubertal period. SVIP staining was similar to the neonatal period. While weak staining was observed in the tubular area, no staining was observed in the peritubular area. Moderate staining with p97/VCP was detected in the prepubertal group. Staining was more pronounced in the tubular area compared to the peritubular area (Figure 1C,D).

The localization of p97/VCP and SVIP in different testicular and interstitial cells in human testicular tissue belonging to the pubertal group was studied in detail. In this group, where the staining intensities of p97/VCP and SVIP were observed to increase intensely, it was noticed that the cell organization in both the peritubular and tubular areas was arranged towards an appearance close to the adult testis. During this period, when the expression of both proteins was prominently seen in the interstitial area, especially in Leydig cells, staining was predominately observed in spermatogonia and spermatocytes belonging to the germ cell line located in the tubules. SVIP staining showed increased expression compared to neonatal and pubertal periods, and the staining intensity for this protein was considered moderate. p97/VCP staining intensity was evaluated as moderate in this period (Figure 1E,F).

In the testis tissues of the adult group, cells belonging to the whole series (spermatogonia, primary and secondary spermatocytes, round and long spermatids), Sertoli cells and sperm in the lumen were observed in the seminiferous tubules. p97/VCP and SVIP immunostaining intensity increased the most in this group. Strong staining was observed with SVIP, and staining was higher in the tubular area than in the peritubular area. Strong p97/VCP staining was observed in both the tubular and peritubular areas in adult testis. Both proteins showed predominant staining in the peritubular area, especially around Leydig cells (Figure 1G,H).

Geriatric group human testis tissues were examined and only spermatogonia were seen in the seminiferous tubules with increased vacuolization. No sperm were seen in the tubules because the germ cell development stopped at the spermatogonia stage. Connective tissue and Leydig cell aggregation were noticeable in the interstitial area around the seminiferous tubules, where the basement membrane was thickened. The number of Leydig cells had also decreased. SVIP and p97/VCP staining decreased in the geriatric group. There was little staining indicating p97/VCP and SVIP expressions in geriatric human testis tissues.

**Figure 2:**
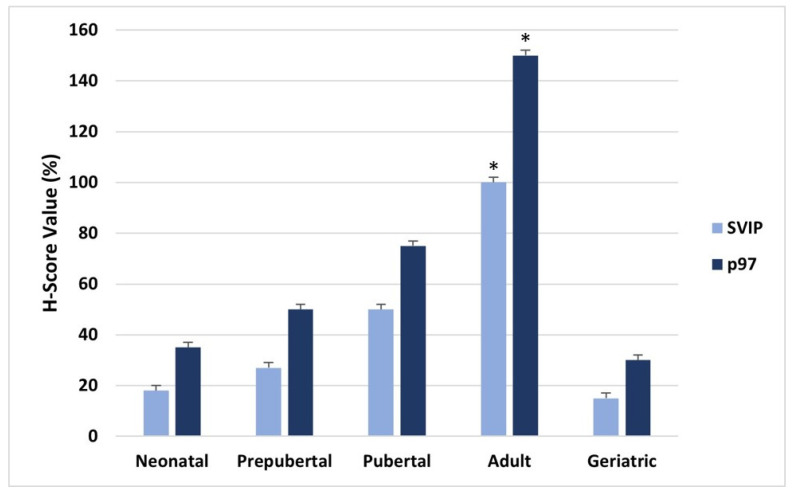
H SCORE values of SVIP and p97/VCP immunostaining intensities in neonatal, prepubertal, pubertal, adult and geriatric testes. The data are represented as mean ± standard of error mean (SEM). * *p* < 0.05, adult versus geriatric testes for SVIP and p97/VCP.

According to the H-SCORE analysis, it was determined that both SVIP and p97/VCP expression in human testicular tissues were correlated with age and increased. The expression of both proteins was found to be significantly decreased in the geriatric age group compared to the adult age group (*p* < 0.05) (Figure 2).

## 4. Discussion

In this study, we investigated the expression of p97/VCP and SVIP proteins in human testis tissue at various postnatal developmental stages. We observed that p97/VCP and SVIP were expressed in neonatal, prepubertal, pubertal, adult and geriatric human testicular tissues, which we evaluated using the immunohistochemical staining method. While the expressions of these proteins were low in the neonatal period, they increased gradually in the prepubertal, pubertal and adult periods. p97/VCP and SVIP protein expressions peaked in the adult period, and a significant decrease was detected in the geriatric period.

p97/VCP and one of the proteins it interacts with, SVIP, are proteins in the ERAD family that are involved in UPS. p97/VCP was described by Moir et al. in 1982 and is also called CDC48 [9,18]. It has an important function in controlling the levels of cellular proteins. It is involved in essential cellular processes such as cell cycle progression, signal transduction, and cell transformation [19]. It has been found in the heart, lung, liver, brain, ovary, breast, and skeletal muscle in humans [5]. It has been reported that p97/VCP plays a role in pathologies such as neurodegenerative disorders (Creutzfeldt-Jakob, Alzheimer’s, Parkinson’s disease), pulmonary pathologies, protein misfolding disorders, Paget’s disease and retinal degeneration [20,21,22,23]. Studies have shown that there is a relationship between increased p97/VCP expression and cancer prognosis and metastatic potential. p97/VCP, which is associated with tumor invasion and metastasis, plays a role in regulating the activity of nuclear factor κB. Nuclear factor κB is a transcription factor involved in cell proliferation, antiapoptosis and invasion. There have been many studies on various types of cancer such as colorectal cancer, breast cancer, prostate cancer, lung cancer, thyroid cancer, and bone cancer. It has been reported that p97/VCP may be a potential biomarker and therapeutic target [8,24,25,26].

SVIP is a protein also involved in ERAD that binds to p97/VCP, an ATPase, and regulates its functions. Nagahama et al. were the first to find that SVIP is a protein that binds to and interacts with p97/VCP. SVIP participates in vacuole formation and autophagy and regulates ERAD inhibition [27].

Throughout the postnatal period, the human testis shows various developmental stages. In the neonatal period, testicular tubules are composed only of gonocytes and somatic cells. In the prepubertal period, spermatogonia begin to appear, and spermatocytes appear at puberty. In the adult testis, elongated spermatozoa are observed in the testicular tubules [28,29]. In our study, we found that the UPS proteins p97/VCP and SVIP were expressed in neonatal, prepubertal, pubertal, adult and geriatric testes. The low expression of these proteins in the neonatal period suggests that the functions of these proteins are not clear or are less significant in the early developmental period. The increase that begins in the prepubertal period reaches its highest level in adulthood. A significant decrease is observed in the geriatric period. This increase in the expression of p97/VCP and SVIP in parallel with testicular growth indicates that p97/VCP and SVIP are among the proteins that may be required for testicular growth. A review of the literature has shown that p97/VCP is an essential protein for cell growth [5,8].

In an experimental study to determine the expression of p97/VCP and Jab1/CSN5, testicular and epididymal tissues from postnatal 5-, 15-, 30- and 60-day-old rats were examined using immunohistochemistry and Western blotting methods. As a result, it was observed that the expression of both proteins increased gradually after birth. Therefore, the authors reported that the gradual increase in the expression of p97/VCP and Jab1/CSN5 proteins is associated with testicular growth and may play an important role in cell proliferation in the testis and epididymis [16].

In another experimental study, the interaction between Smad1, which has different biological functions, and p97/VCP protein, a member of UPS, was investigated during testicular and epididymal development. Testicular and epididymal tissues taken from 5-, 15- and 60-day-old rats were examined using immunohistochemistry, immunofluorescence, Western blot and immunoprecipitation techniques. As a result of the study, they showed that both proteins co-located in spermatogonia, Sertoli, interstitial cells and spermatocytes and there was interaction between them [17]. Signaling molecules and multiple signaling pathways are important in the regulation of spermatogenesis. Elucidating the molecular mechanisms of signaling molecules and pathways is essential for new therapeutic targets for male infertility [28,30].

p97/VCP, which belongs to the Type II AAA ATPase family, consists of a substrate and a cofactor-binding N domain and an ATPase domain called D1 and D2. It shows a hexameric double-ring structure. p97/VCP performs its main function of cleaving protein complexes by unfolding proteins as a ubiquitin-selective chaperone. Its function in many molecular mechanisms that are necessary for cell viability, such as ubiquitin-mediated cell destruction, autophagy, and cell cycle, has not been fully elucidated [9].

Sertoli cells are cells of testicular tissue with important functions. They provide control of spermatogenesis, phagocytize germ cells, release spermatids in spermiogenesis, provide support and manage hormonal regulation [31]. Autophagic mechanisms of the Sertoli cell have been shown to be essential for normal spermatogenesis [32]. At the same time, the disruption of autophagy in Sertoli cells leads to germ cell apoptosis, decreased spermatozoa count, and impaired spermicide [33].

In the study by Cayli et al., which was conducted to evaluate the mechanisms of action of p97/VCP in autophagy and apoptosis in Sertoli cells in mouse testicular tissues, they used a specific inhibitor for P97/VCP. As a result of the experimental study, they stated that the inhibition of p97/VCP function using siRNA and DBeQ impairs autophagosome maturation. They also reported cell cycle arrest and the induction of apoptosis in mouse Sertoli cells [34].

In another study on the autophagy molecular mechanisms of p97/VCP, it was shown that p97/VCP is required in autophagosome maturation steps in normal placenta and these steps are disrupted in preeclamptic placentas [35].

The main functions of the testis are spermatogenesis and androgen production. Androgens are effective at regulating the differentiation of the male sex. Androgens are steroid hormones and include testosterone (T) and dihydrotestosterone (DHT). Through androgen receptors, these hormones have roles in pubertal development, the maintenance of secondary sex characteristics, and sexual maturation [29].

The androgenic hormone testosterone in the steroid structure is produced in Leydig cells in the testis. It is controlled by the Luteinizing hormone (LH) secreted from the pituitary gland. Leydig cells are the main steroidogenic cells of the testis. Steroidogenic enzymes and some other factors are required for the synthesis of testosterone in testicular Leydig cells [11]. Changes in the expression of proteins in the testosterone synthesis steps and low testosterone synthesis amounts may cause sexual dysfunctions and infertility. Lamberts et al. reported a decrease in cellular steroidogenic activity with aging in testicular Leydig cells [36].

p97/VCP is an ERAD-associated protein. SVIP, an adapter ER protein that binds directly to p97/VCP, localizes close to the ER membrane surface. SVIP interacts with p97/VCP and inhibits it [12,37]. ERAD has been reported to be regulated by androgens. Studies have shown that p97/VCP binds to androgen receptors [38]. It has also been identified as an androgen-responsive gene in SVIP, and its expression has been described to be regulated by androgens [37]. SVIP expression has been shown to be androgen-mediated downregulation in prostate cancer cells. A similar situation was observed in glioma cells [38,39]. The androgen response in Sertoli, Leydig and peritubular cells is necessary for testicular developmental steps and normal testicular function. Leydig cells have ER, mitochondria and especially abundant lipid droplets for androgen production [40,41].

In the experimental study by Akcan et al., Leydig cells of postnatal mouse testicular tissues were examined. They investigated the interaction between the SVIP protein and the steroidogenic acute regulatory protein (StAR). As a result of the experiment, they detected SVIP expression predominantly in Leydig cells and other testicular cells. They also demonstrated the localization of SVIP expression to lipid droplet membranes in Leydig cells [42].

In response to androgen production in Leydig cells, changes occur in steroidogenesis-related proteins. In addition, there are changes in the amount and size of lipid droplets [43]. In recent studies, it has been reported that lipid droplets contain a lipid core, phospholipid layer and many proteins [41].

In another study in mouse and human Leydig cell lines, the relationships between p97/VCP, SVIP and ERAD-related proteins were analyzed. Information on the function of p97/VCP and SVIP in testosterone synthesis steps in Leydig cells was obtained. Testosterone levels were found to decrease after the transfection of SVIP and p97/VCP with siRNAs. As a result of the experiment, the authors explained that p97/VCP and SVIP play a role in the steroidogenesis process. The authors suggested that this may be useful for treatments of male infertility and testosterone-related sexual dysfunctions [11].

UPS and autophagy are two pathways that provide protein quality control [44]. They carry out the breakdown and cleaning of damaged proteins. UPS and autophagy have different mechanisms, but they also have similarities. p97/VCP has important roles in both UPS and autophagy [4]. p97/VCP is one of the essential proteins for cell survival [45]. Studies have reported that it is related to the biology of cancer [46].

In the study by Nakkaş et al., the expressions of UPS proteins such as p97/VCP, ubiquitin and Jab1/CSN5 and autophagic proteins such as p62, LC3B and Beclin1 were analyzed with the immunohistochemical method in 120 cases of testicular cancer from orchiectomy materials. Different types of testicular tumor tissues and normal testicular tissues adjacent to the tumor were evaluated. A significant increase in p97/VCP expression was observed in neoplastic testicular tissues compared to normal testicular tissues. The expression of p97/VCP in normal testicular tissues was detected in the cytoplasm of spermatogenic cells and Sertoli cells. In neoplastic testicular tissues, it was seen both in the cytoplasm and nucleus of tumor cells. The researchers concluded that p97/VCP expression is higher in testicular tumors and also translocated from the cytoplasm to the nucleus of tumor cells [47].

The inhibition of the function of P97/VCP is known to cause rapid caspase activation and apoptosis in cancer cells [45]. The discovery of molecules that inhibit P97/VCP is an important step in the treatment of cancer in humans [48].

Our study was performed with the immunohistochemical staining method using human testis tissues to determine p97/VCP and SVIP expressions. Testicular tissues were obtained from paraffin blocks in our archive. Our results are limited to immunohistochemical data. Since frozen testicular tissue samples were not available, analyses with other methods could not be performed.

## 5. Conclusions

We investigated the expression of p97/VCP and SVIP proteins in human testis tissues at different postnatal age periods using the immunohistochemical method. We observed the expression of both proteins in all age periods. The expression increased gradually from the neonatal period to adulthood, and after peaking in adulthood showed a significant decline in the geriatric period.

The long-term aim of our study is to use these proteins as testicular cell markers in unexplained infertility cases. In recent years, studies to explain male infertility have often focused on molecular mechanisms. We think that our study may contribute to further studies on the use of these proteins as testicular cell biomarkers in unexplained male infertility cases. Further research is needed in the future to fully elucidate the molecular mechanisms of testicular tissue.

## Data Availability

The datasets generated and analyzed during this study are not publicly available due to a lack of participants’ agreement.
